# Cell type-dependent Erk-Akt pathway crosstalk regulates the proliferation of fetal neural progenitor cells

**DOI:** 10.1038/srep26547

**Published:** 2016-05-23

**Authors:** Ji heon Rhim, Xiangjian Luo, Dongbing Gao, Xiaoyun Xu, Tieling Zhou, Fuhai Li, Ping Wang, Stephen T. C. Wong, Xiaofeng Xia

**Affiliations:** 1 Chao Center for BRAIN, Department of Systems Medicine and Bioengineering, Houston Methodist Research Institute, Houston, TX 77030.; 2 Weill Cornell Medical College, Cornell University, New York, NY 10065; 3 Department of Pathology and Genomic Medicine, Houston Methodist Research Institute, Houston, TX 77030.

## Abstract

Neural progenitor (NP) cells are the multipotent cells that produce neurons and glia in the central nervous system. Compounds regulating their proliferation are key to both understanding brain development and unlocking their potential in regenerative repair. We discuss a chemical screen that unexpectedly identified inhibitors of Erk signaling potently promoting the self-renewing divisions of fetal NP cells. This occurred through crosstalk between Erk and Akt signaling cascades. The crosstalk mechanism is cell type-specific, and is not detected in adult NP cells as well as brain tumor cells. The mechanism was also shown to be independent from the GSK-3 signaling pathway, which has been reported to be a major regulator of NP cell homeostasis and inhibitors to which were also identified in the screen. *In vitro* Erk inhibition led to the prolonged rapid expansion of fetal NP cells while retaining their multipotency. *In vivo* inhibitor administration significantly inhibited the neuronal differentiation, and resulted in increased proliferative progenitor cells in the ventricular/subventricular zone (VZ/SVZ) of the embryonic cortex. Our results uncovered a novel regulating pathway for NP cell proliferation in the developing brain. The discovery provides a pharmacological basis for *in vitro* expansion and *in vivo* manipulation of NP cells.

The three major cell types of the mammalian brain, namely neurons, astrocytes, and oligodendrocytes, are derived from a common ancestor called the NP cell that originally arises from the neural plate in the early embryo^1^,^2^. During brain development, exquisite coordination between NP cell self-renewing proliferation and differentiation eventually produces all the neuronal and glial cells that populate the mature brain. How the balance between the self-renewal and differentiation is achieved is not entirely clear. Many extrinsic factors have to be involved to achieve the subtle temporal and spatial control, including Wnts^3^, fibroblast growth factors (FGFs), epidermal growth factor (EGF)^4^, Sonic Hedgehog (SHH)^5^,^6^, bone morphogenetic protein (BMP)^7^, and Notch ligands^8^. The intrinsic factors that mediate their effects have started to emerge recently^9^,^10^. Notably, intrinsic factors, such as the GSK-3s^9^, are often positioned at the converging node of several extrinsic signals, to coordinate the self-renewal and differentiation balance. The discovery of such critical node molecules, especially the ones in which small molecule compounds can intervene^11^, is key to both understanding brain development and unlocking the potential of NP cells in regenerative repair^12^.

In an attempt to obtain a more comprehensive profile of the regulating network and identify effective chemical probes, we carried out chemical genetic screening^13^ for compounds promoting the self-renewing proliferation of fetal NP cells. Our results unexpectedly identified Erk signaling inhibitors (ERKi) to be among the most potent pharmacological classes. Further experiments showed its effect to be via Erk-Akt crosstalk, to release the cell cycle arrest, and inhibit neuronal differentiation. The mechanism is independent of GSK-3 signaling and represents a novel key node coordinating the NP cell self-renewal and differentiation balance. Lastly, we demonstrate the application of ERKi in both *in vitro* NP cell culture and *in vivo* NP cell manipulation in the developing brain.

## Results

### Major pharmacological classes promoting fetal NP cell proliferation

**Figure 1:**
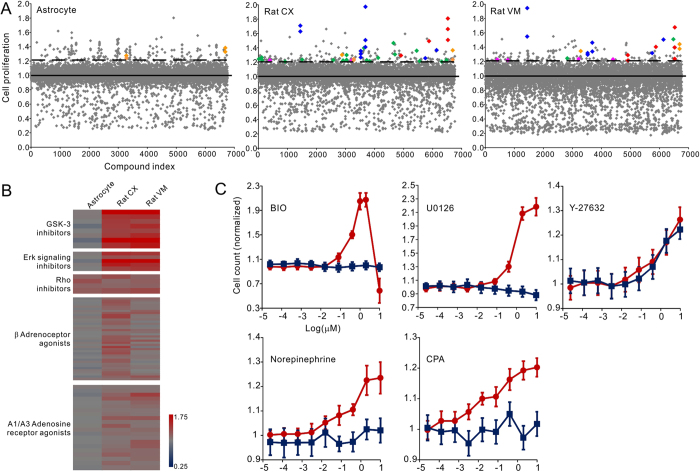
Chemical genetic screen of the proliferation of fetal NP cells. (**A**) Scatter plot of all the compounds screened on the two types of fetal NP cells and the control astrocyte cells. Five pharmacological classes were identified to increase cell proliferation above the 3σ threshold (dashed lines) from the control mean (solid lines). Blue, GSK3i; red, ERKi; green, β adrenoceptor agonists; pink, A1/A3 adenosine receptor agonists; orange, RHOi. (**B**) Proliferation assay results of a comprehensive collection of the five pharmacological classes. Results were normalized and presented in heat maps. Consistent promoting effects were observed for the structurally diverse compounds. The effects of ERKi and GSK3i were the strongest. (**C**) Dose-response curves (mean ± s.d., n = 3) of GSK3i BIO, ERKi U0126, RHOi Y27632, β adrenoceptor agonist norepinephrine (NE), and A1/A3 adenosine receptor agonist cyclopentyladenosine (CPA). The results on Rat CX cells were shown in red and control astrocytes shown in blue. ERKi and GSK3i were confirmed to be the two most potent classes of chemicals. Except for RHOi, the promoting effects of the other four chemicals were not detected on the control astrocytes.

To facilitate the screen of enhanced proliferation, we used primary cultured fetal rat NP cells, which gradually become dormant *in vitro*^14^. To circumvent the possible regional discrepancy, we tested the NP cells from both the fetal rat cortex (Rat CX) and ventral mesencephalon (Rat VM), using the rat primary astrocytes as a control (Supplementary Fig. 1). During the screen, the undifferentiated cells were grown in laminin-coated 384-well plates and treated with a 2-μM compound for 72 hours^15^. Cell proliferation was measured by the incorporation of the tetrazolium dye (MTS). Over 5,000 bioactive compounds were tested 2 to 4 times, and the mean of the results (Supplementary Table 1) was used to select the primary hits, which were defined as compounds that induce proliferation at least three standard deviations (3σ) above the vehicle control mean (Fig. 1A). Clustering of the hits revealed five pharmacological classes including GSK-3 inhibitors (GSK3i), ERKi, Rho and associated kinase inhibitors (RHOi), β adrenoceptor agonists, and A1/A3 adenosine receptor agonists. To confirm the results and exclude the off-target possibilities, we composed a list of structurally diverse compounds for each class (Supplementary Table 2) and tested them on the NP cells. To avoid the possible artifact of MTS assay^16^, we directly counted the total cell numbers after three days of treatment as a surrogate marker of cell proliferation. Consistent promoting effects were observed for various members of the five pharmacological classes (Fig. 1B), supporting the effects to be target specific. Titration of selected examples from the five classes on Rat CX cells showed that they function in a dose-dependent manner with EC_50_ at sub-μM to μM range (Fig. 1C). The highly proliferative compound treated cells retained tripotency and can be differentiated into neurons, astrocytes, and oligodendrocytes (Supplementary Fig. 2). Except for the RHOi, the promoting effects were selective for fetal NP cells and not detected in the control astrocyte cells (Fig. 1B,C). Since no difference was detected between the Rat CX and Rat VM cells in all of the tests, only the results on Rat CX cells were shown hereafter.

### ERKi promote fetal NP cell proliferation through Erk-Akt crosstalk

**Figure 2:**
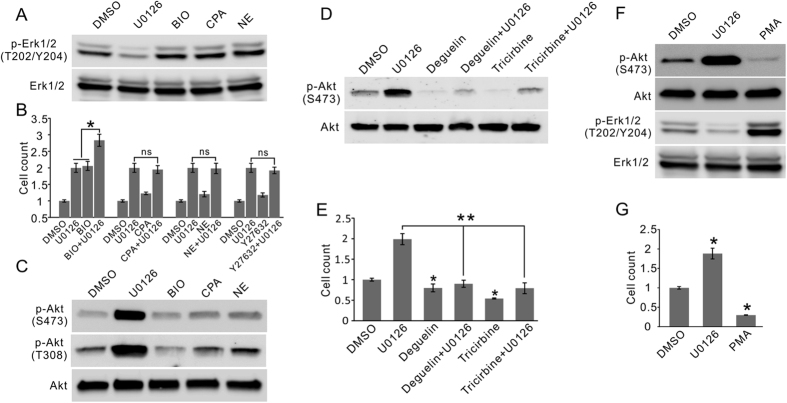
Signaling pathways mediating the effect of fetal NP cell proliferation promoting compounds. (**A**) None of the chemicals significantly promoted Erk1/2 phosphorylation. ERKi U0126 as expected downregulated Erk1/2 phosphorylation. (**B**) Co-administration of U0126 markedly increased the effect of GSK3i BIO but had no effect on the other chemicals. Mean ± s.d., n = 4. (**C**) Short-term (15 minutes) U0126 treatment led to a marked increase in Akt phosphorylation at both S473 and T308. Increase in Akt phosphorylation was also detected in cells treated with A1/A3 adenosine receptor agonists CPA and β-adrenoceptor agonist NE. (**D**) The effect of U0126 on Akt phosphorylation was significantly inhibited by Akt inhibitors Deguelin (20 nM) or Tricirbine (500 nM). (**E**) The effect of U0126 on the fetal NP cell proliferation was also significantly inhibited by Deguelin or Tricirbine. Mean ± s.d., n = 4. (**F**) PMA (1 μM) promoted Erk1/2 phosphorylation but inhibited Akt phosphorylation in fetal NP cells. (**G**) PMA adversely regulated fetal NP cell proliferation. Mean ± s.d., n = 4. *P < 0.05. **P < 0.01. All gels were run under the same experimental condition. Cropped images were shown in the figure. Full length blots are included in the Supplementary Information.

The compounds identified in the screen likely reflect the major regulating mechanisms of the NP cell self-renewal and proliferation. While the results are mostly consistent with the literature^9^,^17^,^18^,^19^, the finding of ERKi being the strongest potentiator was unexpected. Time-lapse imaging clearly recorded a marked increase in mitosis events as the result of Erk inhibition (Supplementary Movie 1), proving a direct role of the signaling in NP cell cycle. The Erk signaling cascade is a chief driver of cellular proliferation in response to extracellular stimuli such as the growth factors^20^,^21^,^22^. Accordingly, it has been suggested to drive the effect of several NP cell-stimulating molecules^18^,^23^,^24^,^25^. Consequently, inhibition of the pathway was expected to attenuate cell proliferation. To solve this contradiction, we first examined the level of Erk signaling in fetal NP cells treated with the five promoting compounds. None of the chemicals significantly increased Erk1/2 phosphorylation, while the ERKi U0126 predictively reduced it (Fig. 2A), excluding the signaling itself to be the major mediator of the proliferation-promoting effects. Moreover, if Erk signaling was the major mediator, its inhibition should abolish the effect. However, when U0126 was co-administered with the other four compounds, no inhibitory effect was detected (Fig. 2B). In contrast, the combination of U0126 with BIO resulted in a further marked increase compared to either chemical alone (Fig. 2B). Further testing consistently showed that the combination effect exceeded the saturation effect of either compound on fetal NP cells cultured in various prevailing methods^26^ (Supplementary Fig. 3), suggesting that these two most potent compounds achieve the proliferation promoting effect through mechanisms that are independent of each other.

To identify the molecular pathway that accounts for the proliferation-promoting effect of ERKi, we examined the Akt signaling cascade that frequently interacts with the Erk signaling pathway^27^. A dramatic increase in Akt phosphorylation at both S473 and T308 was detected upon Erk inhibition, and increases were also observed in cells treated with β-adrenoceptor and A1/A3 adenosine receptor agonists (Fig. 2C), suggesting a primary role for Akt instead of Erk in directly mediating the proliferation enhancing effect, and a possible role for the Erk-Akt crosstalk to account for the ERKi effect. To test the hypothesis we treated the Rat CX cells with two Akt inhibitors Deguelin and Tricirbine. Both compounds effectively blocked the phosphorylation of Akt (Fig. 2D), and consequently reduced cell proliferation (Fig. 2E), although no toxicity was detected and the treated cells retained healthy morphology (Supplementary Fig. 4). Moreover, both inhibitors were able to significantly inhibit the effect of U0126 on Akt phosphorylation (Fig. 2D), concurrently the marked proliferation enhancing effect of U0126 was abolished in the presence of the two inhibitors (Fig. 2E). Another compound Phorbol 12-myristate 13-acetate (PMA), which preferentially activates the Erk pathway as opposed to the Akt pathway, promoted Erk1/2 phosphorylation but inhibited Akt phosphorylation, and consequently adversely regulated fetal NP cell proliferation (Fig. 2F,G), further supporting the role of Erk-Akt crosstalk in regulating fetal NP cell proliferation.

### ERKi and GSK3i independently regulate fetal NP cell proliferation

**Figure 3:**
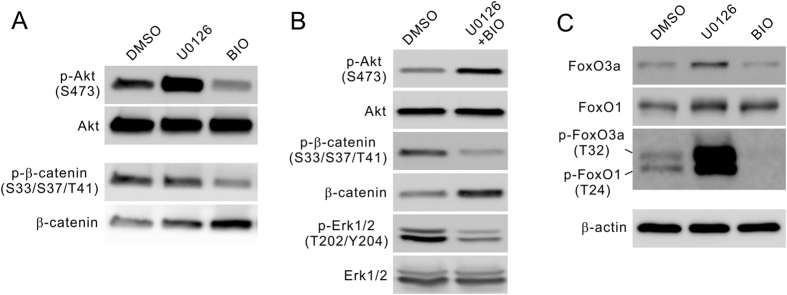
ERKi and GSK3i regulate NP cells through different pathways. (**A**) ERKi and GSK3i differently regulate PI3K/Akt and β-catenin pathways. U0126 increased while BIO decreased Akt phosphorylation after 24 hours of treatment. BIO prevented β-catenin phosphorylation leading to intact β-catenin accumulation, while U0126 only had a marginal effect. (**B**) Combined U0126/BIO treatment led to both the enhancement of Akt phosphorylation and the increase of intact β-catenin by preventing its phosphorylation. The phosphorylation of Erk1/2 was decreased by the combination treatment and therefore is not responsible for the proliferation promoting. (**C**) U0126 markedly increased the phosphorylation of Akt downstream targets FOXO1 and FOXO3a, while BIO decreased it. All gels were run under the same experimental condition. Cropped images were shown in the figure. Full length blots are included in the Supplementary Information.

GSK-3 has been proposed as a master regulator of NP cell homeostasis^9^. Our results that ERKi and GSK3i can both dramatically promote fetal NP cell proliferation and their effects are additive would suggest Erk-Akt crosstalk to be an additional independent major regulatory mechanism. GSK-3 inhibition promotes NP cell proliferation through Wnt signaling (Fig. 3A)^9^, and clearly not Akt signaling, as short-term BIO treatment had little effect on Akt phosphorylation while long-term treatment caused decreasing (Fig. 3A). The promoting effect of ERKi is not likely to be through the β-catenin signaling entirely. Although U0126 treatment did increase β-catenin accumulation possibly as a result of the Akt effect (Fig. 3A), the increase was much lower than BIO and could not explain their similar stimulating effect on the fetal NP cell proliferation. Combination of BIO and U0126 treatment was able to both significantly enhance Akt phosphorylation and intact β-catenin accumulation (Fig. 3B), thereby further increase the cell proliferation. Again Erk signaling was shown to be unlikely involved in the proliferation promotion, as the phosphorylation level of Erk1/2 was decrease by the combination treatment (Fig. 3B).

When the possible Akt downstream targets were tested, we detected marked enhancement in the phosphorylation of the Forkhead family transcription factors FoxO1 and FoxO3a (Fig. 3C), which have been reported to regulate the pool size of NP cells^28^. The phosphorylation would result in the inactivated forms of the transcription factors and release cell cycle inhibition, to account for proliferation enhancing. The FoxO1/3a mechanism is not utilized by GSK3i, which actually reduced their phosphorylation possibly as a result of dampened Akt signaling (Fig. 3C). Together these results confirmed that Erk inhibition is an independent novel mechanism promoting NP cell proliferation.

### ERKi can be used effectively for *in vitro* NP cell culture and *in vivo* NP cell manipulation

**Figure 4:**
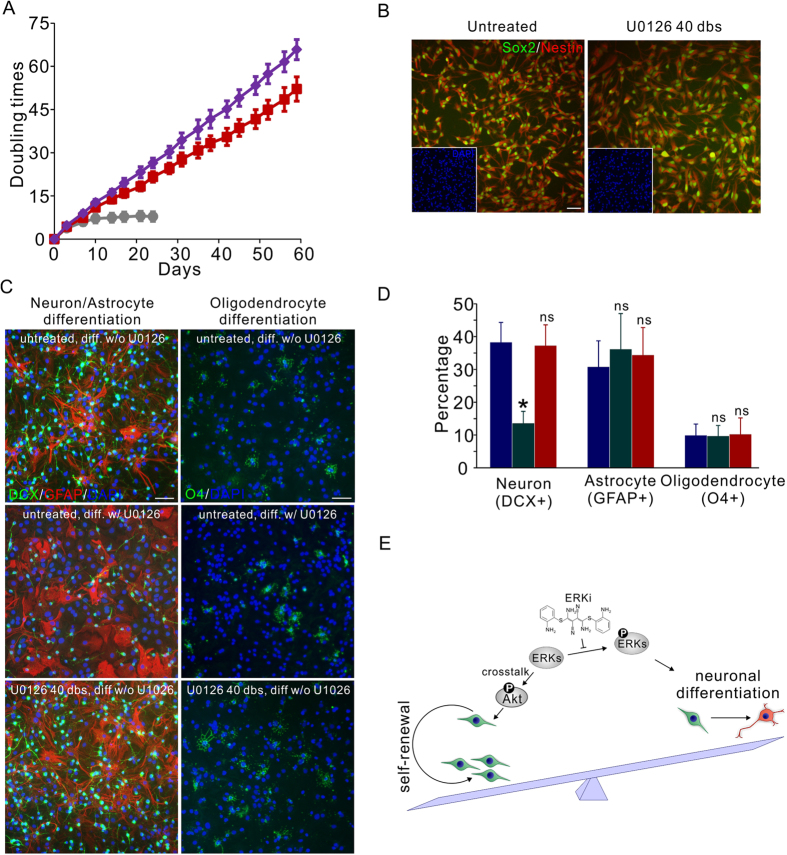
ERKi regulates the self-renewal and differentiation balance of NP cells *in vitro*. (**A**) Continuous ERKi treatment (3 μM U0126) led to the prolonged expansion of monolayer cultured Rat CX cells at a stable doubling rate of about 27 hours (red). Combination with GSK3i (1 μM BIO) further reduced the cell cycle time to 22 hours (purple). Untreated cells suffered significant cell cycle arrest along with expansion (grey). Mean ± s.d., n = 3. (**B**) Rat CX Cells expanded with ERKi for 40 doublings (dbs) retained homogeneous expression of NP cell markers Sox2 and Nestin. (**C**) After ERKi removal, the ERKi treated cells can be differentiated into neurons (DCX+), astrocytes (GFAP+) and oligodendrocytes (O4+) (bottom, U0126 40 dbs, diff w/o U0126), indistinguishably from the untreated cells (top, untreated, diff w/o U0126). The presence of ERKi during differentiation significantly inhibited the neuronal differentiation but not glial differentiation (middle, untreated, diff w/ U0126). Scale bars, 50 μm. (**D**) Quantification of the percentages of neurons, astrocytes and oligodendrocytes generated by untreated Rat CX cells differentiating without U0126 (blue); untreated Rat CX cells differentiating with 3 μM U0126 (green); and Rat CX cells treated with U0126 for 40 dbs differentiating without U0126 (red). Mean ± s.d., n = 6. *P < 0.05, ns, not significant. (**E**) A model summarizing the effect of ERKi compounds on fetal NP cells. Erk signaling is a key node regulating the balance between the self-renewal and neuronal differentiation of fetal NP cells. By inhibiting Erk phosphorylation, the Akt pathway is activated via Erk-Akt crosstalk, shifting the balance toward the self-renewing proliferation of fetal NP cells.

Adherent culture of fetal rat NP cells suffered from progressive cell cycle arrest^14^,^29^, and a gradual switch from neurogenic to gliogenic^30^. When the Rat CX cells were cultured on laminin-coated surfaces, in less than 10 doublings the cell expansion was markedly reduced (Fig. 4A). This has severely limited the availability of high-fidelity NP cells and impeded the enthusiasm of using these cells in pharmacological screening. The discovery of major regulating pathways of NP cell self-renewing proliferation provided solutions to circumvent the problem. Persistent ERKi treatment effectively prevented cell cycle arrest, leading to the prolonged stable expansion of Rat CX cells in monolayer culture. In our experiment, cells were continuously passaged for more than 40 doubling times in the presence of 3 uM U0126. The treatment resulted in a stable cell doubling time of about 27 hours (Fig. 4A). Consistent with its independence from GSK-3 signaling, combining 1 uM BIO with 3 uM U0126 further shortened the doubling time to about 22 hours (Fig. 4A). To date, slowed proliferation has not been observed. Homogeneous expression of NP cell markers including Sox2 and Nestin were retained in the long-term inhibitor treatments (Fig. 4B). To test how ERKi affects cell differentiation and whether multipotency is retained after prolonged ERKi treatment, Rat CX cells were differentiated after expansion for over 40 doublings. While the presence of ERKi strongly suppressed the neuronal differentiation (Fig. 4C,D), the differentiation capacity could be fully released upon inhibitor removal, leading to the generation of neurons, astrocytes, or oligodendrocytes at comparable efficiency to low-passage untreated Rat CX cells (Fig. 4C,D). Moreover, after the removal of U0126, the global gene expression of the treated cells were largely unchanged compared to the untreated low passage cells. Microarray analysis showed that only 39 (0.14%) genes were changed by more than 2-fold with p values less than 0.05 (Supplementary Table 3, for raw data please see Supplementary Table 4). The changed genes were not strongly associated in function. Only seven of the genes were clustered in two groups using DAVID gene function classification tool when the stringency was set at the lowest^31^,^32^, and in both groups no annotation directly associated with the function of NP cells was detected (Supplementary Fig. 5). Expressions of the NP cell biomarkers such as Sox2 and Nestin, and the genes known to be important for the neurogenesis such as the bHLH family transcription factors, or oligogenesis such as Olig2 and Notch signaling genes were not altered in the ERKi maintained cells (Supplementary Table 4). Taken together these results suggest that ERKi treatment maintained the self-renewal proliferation of fetal rat NP cells *in vitro*. With the prolonged self-renewal and tripotent capacity the culture technique offers critical opportunities for pharmacological screening and related *in vitro* applications^33^. These experiments using ERKi compound as a probe also revealed the critical role of Erk signaling in maintaining the balance between the self-renewal and differentiation in fetal rat NP cells: activated Erk signaling promotes the NP cell neuronal differentiation. Upon inhibition of the pathway, the neuronal differentiation is suppressed and the Akt pathway is activated via Erk-Akt crosstalk, resulting in the phosphorylation of FOXO proteins and the release of cell cycle inhibition, therefore the cells are switched to continuous self-renewing proliferation (Fig. 4E).

**Figure 5:**
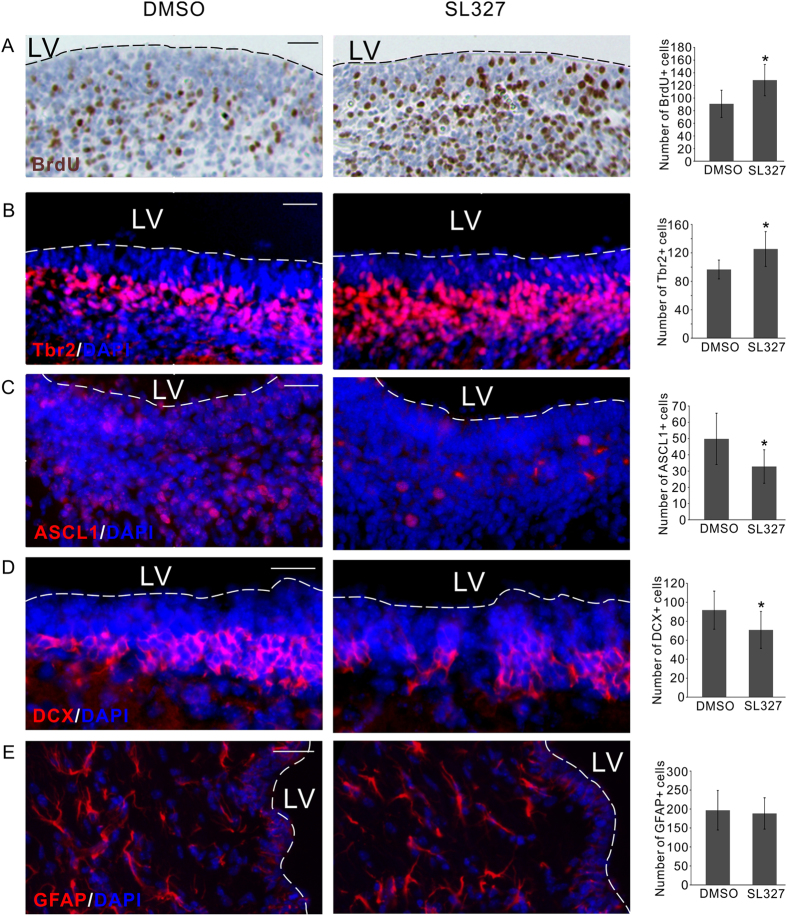
ERKi regulates NP cells i*n vivo* in the developing rat brain. (**A**) Injection of brain permeable SL327 compound resulted in more BrdU+ proliferative cell in the VZ/SVZ of the embryonic brain. (**B**) The number of Tbr2+ intermediate progenitor cells was also significantly increased by SL327 injection. (**C**) The number of ASCL1+ cells was significantly reduced by SL327 injection. (**D**) The number of DCX+ neuroblasts was also significantly reduced by SL327 injection. (**E**) The number of GFAP+ astrocytes was not affected by SL327 injection. In all the panels representative sections were shown in the left two images, the quantification of the number of positive cells was shown in the right bar graph. All bar graphs represent Mean ± s.d., n = 6. LV, lateral ventricle. *P < 0.05. Scale bars, 50 μm.

To test the effect of ERKi *in vivo* we injected pregnant rat with brain permeable SL327 compound and examined its effect on the neonatal brains (P0). Consistent with the result *in vitro*, BrdU incorporation experiment indicated a significant increase of active cell proliferation as a result of ERKi administration *in vivo* (Fig. 5A). When the responding cell types were examined, we noticed that the number of neural progenitor cells expressing Tbr2 significantly increased (Fig. 5B). Consistent with the inhibitory effect of ERKi on the neuronal differentiation *in vitro*, SL327 injection reduced the number of progenitor cells expressing ASCL1 (Fig. 5C), which is known to promote cell cycle exit and neuronal differentiation of NP cells^34^,^35^. As a result the number of DCX expressing neuroblasts was also significant reduced (Fig. 5D). The glial differentiation was not affected by the SL327 injection as indicated by the unchanged number of GFAP+ cells (Fig. 5E). Taking together, our result consistently showed that ERKi can be used as an effective pharmacological intervention to increase the proliferation of NP cells both *in vitro* and *in vivo*.

### The proliferation-promoting effect of ERKi is selective to fetal NP cells

**Figure 6:**
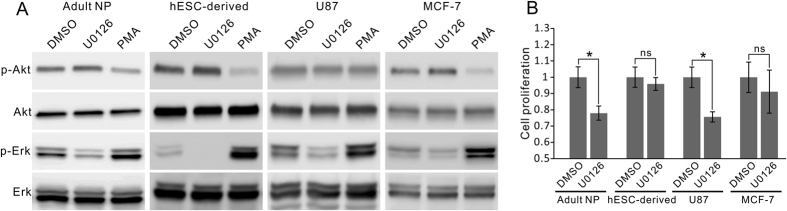
Erk-Akt crosstalk and the effect of ERKi on proliferation in other cell types. (**A**) ERKi U0126 failed to trigger significant Erk-Akt crosstalk in adult NP cells, hES-derived NP cells, brain tumor U87 cells, and breast cancer MCF-7 cells in which Erk-Akt crosstalk has been extensively studied. The opposite crosstalk can be triggered by PMA in adult NP, hES-derived, and MCF-7 cells. (**B**) U0126 treatment failed to promote the proliferation of the four cell types. An inhibitory effect was observed in adult NP and U87 cells. Mean ± s.d., n = 4. *P < 0.05. ns, not significant. All gels were run under the same experimental condition. Cropped images were shown in the figure. Full length blots are included in the Supplementary Information.

Mechanisms of Erk-Akt crosstalk can significantly differ with the cell types^27^. As opposed to cancer cells^36^, the crosstalk in normal cells is reported less and has not been correlated with normal physiological functions. The current result uncovered a novel cross-inhibition mechanism in fetal NP cells that is utilized in controlling the self-renewing proliferation. Interestingly, this mechanism is highly selective to fetal rat NP cells which undergo progressive cell cycle arrest, and not detected in other cell types that constantly expand *in vitro*, including its adult counterpart^37^, the malignant brain tumor cells, and the NP cells derived from human embryonic stem cells (hESC-derived)^38^,^39^ (Supplementary Fig. 6). Despite the presence of Erk-Akt integration in these cells, as the elevation of the Erk pathway by PMA dampened the Akt pathway, only very weak or no enhancement of Akt phosphorylation is detected in ERKi treated cells (Fig. 6A). Consequently, ERKi failed to increase the proliferation rate of these cells (Fig. 6B).

## Discussion

We report here the major pharmacological classes identified by a chemical genetic screen that promote the self-renewing proliferation of fetal NP cells. The screen result provided the basis for profiling the signaling network that governs the self-renewal of fetal NP cells^13^. As demonstrated in the current study using the unexpected finding of ERKi as a probe, we successfully uncovered a cell type selective Erk-Akt crosstalk pathway, as a novel major regulating mechanism to promote the self-renewing proliferation of fetal NP cells both *in vitro* and *in vivo*^14^,^40^. Our finding provided an effective chemical method to circumvent the challenge of long term *in vitro* expansion of fetal NP cells, as well as a pharmacological strategy to manipulate the NP cells in the developing brain.

Erk and Akt signaling are the two prime controllers of cell proliferation. Molecules enhancing cellular proliferation often induce increased phosphorylation of both Erk and Akt^18^,^23^,^24^,^25^. Our current result clearly confirms that Akt, but not Erk, is directly responsible for driving fetal NP cell proliferation. Akt was previously consistently shown to mediate the effect of other agents specifically promoting NP cell proliferation^41^; and negative regulators of Akt, eg, PTEN, has been shown to attenuate NP cell self-renewal^42^.

Erk and Akt rarely act independently and crosstalk between them is frequent. Interestingly, the crosstalk mechanism is highly variable; both cross-inhibition and cross-activation can possibly happen depending on the cell types^27^. Our results reveal that crosstalk in the fetal NP cell occurs in a cross-inhibition manner, and plays an essential physiological role in regulating its self-renewing proliferation. Such a crosstalk mechanism is advantageous in maintaining the stem cell self-renewal/differentiation balance. Our results (Fig. 4C,D) show that Erk signaling is also involved in the neuronal differentiation of NP cells; others have shown that ERKs play essential roles in the neurogenesis as well as the survival of differentiated neuronal cells^43^,^44^. Collectively these data suggest that Erk is positioned at a converging node of pathways controlling the self-renewal and neuronal differentiation of NP cells, similar to but independent of the GSK3s^9^, serving as a switch to shift the self-renewal/differentiation balance. The existence of multiple independent nodes is necessary, as such to provide flexibility to achieve the exquisite temporal and spatial control by responding to a diversity of extrinsic factors. Our proposed function of Erk in NP cells is consistent with the findings in embryonic stem (ES) cells, in which Erk1/2 has been known to promote cell differentiation through Klf4 phosphorylation^45^, and consequently inhibition of the pathway sustains ES cell self-renewal^46^.

The critical role of Erk for brain development has clearly been demonstrated by studies in knockout animals^21^,^22^,^47^, as well as in human patients carrying the mutations of Erk pathway genes. However, its exact function in neural cells appears to be complex. Both the loss^48^ and gain^49^,^50^ of function mutations of Erk elements result in similar clinical syndromes that have been collectively termed “neuro-cardio-facial-cutaneous (NCFC) syndromes, characterized by thinner cortex and reduced cerebral volume. Our results provided new insights into the cellular mechanism of the disease symptom, and suggest that both the loss and gain of Erk function may lead to the loss of NP cell balance, resulting in either impaired neurogenesis or an insufficient NP cell pool to affect cortex development.

## Methods

### Cell isolation and culture

Animal experiments were carried out in accordance with the protocol approved by the Institutional Animal Care and Use Committee of Houston Methodist Research Institute. Primary fetal NP cells were cultured from the E18 rat brain cortex, or the ventral mesencephalon from the E14 rat embryos. Tissues were dissected and dissociated with accutase (StemCell Technologies, Vancouver, Canada) to single cells, and grown in suspension to form neurospheres first. The spheres were then digested with accutase to single cells and grown adherently as monolayer on 20 μg/ml laminin (Life Technologies, Grand Island, NY, USA) coated surface, and the cells were designated as Passage 1 (P1). For expansion cells were seeded at 1.6 × 10^4^ cells/cm^2^ in laminin coated flasks and passaged every 3 to 4 days. In all the screening and validation experiments, P3 cells were used unless otherwise specified. Multipotent adult NP cells were isolated from the SVZ of 15-weeks old rats, and cultured similarly as fetal NP cells. Briefly, the SVZ was dissected and dissociated with accutase to single cells. The single cells were cultured in suspension to form neurospheres, which were then dissociated to single cells and expanded in monolayer on laminin coated surface and designated as P1 cells. Again P3 cells were used in the experiments. Human embryonic stem cell derived neural stem cells (hES-derived) was purchased from Life Technologies (Life Technologies, Grand Island, NY, USA), which was generated from the NIH approved H9 (WA09) human embryonic stem cells. All the NP cells were cultured in medium consisting of Knockout DMEM/F12 and Stempro neural supplement (Life Technologies, Grand Island, NY, USA), with 10 ng/ml fibroblast growth factor 2 (bFGF) and 10 ng/ml epidermal growth factor (EGF) (StemCell Technologies, Vancouver, Canada). As indicated in the text, primary fetal NP cells were also cultured in semi-attached condition on surfaces coated with 0.2% gelatin (Sigma-Aldrich, St Louis, MO, USA), or in suspension as neurospheres in ultra-low attachment flasks (Corning, Tewksbury, MA, USA).

The control rat primary cortical astrocytes from E19 fetal rat were purchased from Life Technologies ((Life Technologies, Grand Island, NY, USA). Cells were cultured in DMEM with 15% fetal bovine serum, and expanded once before the P2 cells were used in the experiments.

### Chemical Libraries

The compounds libraries used in this study included: the LOPAC library consisting of 1,280 pharmacologically active compounds (Sigma-Aldrich, St Louis, MO, USA); the Enzo Screen-Well kinase inhibitor library consisting of 80 kinase inhibitors; the Enzo Screen-Well Bioactive lipid library consisting of 190 bioactive lipids (Enzo Life Sciences, Farmingdale, NY, USA); the Tocriscreen Mini library consisting of 1,120 biologically active compounds (Tocris Bioscience, Bristol, UK); the Spectrum Collection consisting of 2,320 compounds (MicroSource, Gaylordsville, CT, USA); the Prestwick Chemical library consisting of 1,200 compounds; the Prestwick Phytochemicals Library consisting of 320 phytochemical compounds; the Prestwick Natural Compounds Library consisting of 240 natural compounds (Prestwick Chemical, Parc dinnovation, France). For all libraries the stock solution is 10 mM dissolved in DMSO. Before the screen experiment they were freshly pre-diluted 1:50 in PBS buffer.

### High-throughput screen

Primary NP cells were arrayed into laminin coated 384-well plates at a density of 1,250 cells/well (1.25 × 10^4^ cells/cm^2^) in 100 μl medium using a MultiFlo dispenser (BioTek, Winooski, VT, USA). Then 1 μl pre-diluted compound was transferred from the library plate to the cell plate using a Biomek FX robot (Beckman Coulter, Indianapolis, IN, USA) to reach a final concentration of 2 μM. Cells were grown with the compound for 72 hours before they are assayed using the MTS method (Promega, Madison, WI, USA). Each compound was tested from two to four times and the results were averaged. For control experiment the same screen was carried out on rat primary cortical astrocytes.

### Cell proliferation assays

Cell proliferation was measured using three different methods including MTS assay (Promega, Madison, WI, USA), BrdU ELISA assay (Cell Signaling, Danvers, MA, USA) and nuclei counting. Primary NP cells were seeded into laminin coated 96-well plates at a density of 4 × 10^3^ cells/well (1.25 × 10^4^ cells/cm^2^). Cells were treated with indicated compound or DMSO control for 72 hours before the assays. For MTS assay 20 μl reagent was added to each well and incubated for 3 hours at 37 °C before the absorbance at 490 nm (A490) was measured. For BrdU assay the cells were chased with 10 μM BrdU in the last 4 hours and then fixed, stained with BrdU antibody and then HRP-conjugated secondary antibody, and developed with TMB substrate to measure A450. For nuclei counting the fixed cells were stained with Hoechst 33342 dye (Life Technologies, Grand Island, NY, USA). The plate was scanned with an ImageXpress Micro microscope (Molecular Devices, Sunnyvale, CA, USA) using a 4× objective to take an image covering the whole well for each well. Cell numbers were counted using the ImageJ software with an automatic nuclei counter plug-in.

### Time-lapse imaging

Primary NP cells were seeded into laminin coated 96-well plate at a density of 4 × 10^3^ cells/well (1.25 × 10^4^ cells/cm^2^), in medium containing 3 μM U0126 or equal volume of DMSO vehicle. After 8 hours the cells were fully attached and the plate was transferred into a IncuCyte microscope (Essen Bioscience, Ann Arbor, MI, USA) and bright field images were taken with a 20× objective every 15 minutes for about two and half days.

### Cell differentiation and immunofluorescence staining

For differentiation the NP cells were cultured in laminin coated 96-well plates at 3.5 × 10^4^ cells/cm^2^ with 10 ng/ml bFGF. The next day the culture medium was changed to the neural differentiation medium consisting of neurobasal, B27 supplement and GlutaMax (Life Technologies, Grand Island, NY, USA). After that half of the medium was changed every two days. For the neuron/astrocyte differentiation cells were differentiated for two weeks, then fixed and stained with anti-DCX antibody (Cell Signaling, Danvers, MA, USA); anti-β-tubulin type III (Tuj) antibody (Abcam, Cambridge, MA, USA); or anti-GFAP antibody (Millipore, Billerica, MA, USA). For the oligodendrocyte differentiation cells were differentiated for four weeks, then fixed and stained with anti-O4 antibody (R&D Systems, Minneapolis, MN, USA). For the dopaminergic neuronal differentiation cells were differentiated for four weeks in the presence of 10 ng/ml BDNF and 10 ng/ml GDNF (PeproTech, Rocky Hill, NJ, USA), then fixed and stained with anti-TH antibody (Cell Signaling, Danvers, MA, USA). Fluorescence images were taken using an IX81 inverted microscope (Olympus, Tokyo, Japan). Image quantification was performed using the ImageJ software.

### Gene expression microarray analysis

Gene expression microarray analysis was done by SeqWright (SeqWright, Houston, TX, USA). Total RNA was extracted from cells using a EZ1 RNA Cell Mini Kit (Qiagen, Valencia, CA, USA). Cells were expanded in U0126 for 20 passages and U0126 was removed from medium 48 hours before RNA extraction. The control cells are DMSO treated P2 cells and DMSO was also removed from medium 48 hours before RNA extraction. The RNA concentration was determined with a Nanodrop ND-1000 Spectrophotometer and RNA quality was verified with an Agilent 2100 Bioanalyzer using a RNA Nano Chip. All RNA samples displaying no visible degradation in the Bioanalyzer analysis with two sharp ribosomal peaks. Affymetrix’s GeneChip IVT Express kit was used for cDNA synthesis using 250 ng of total RNA and for *in vitro* transcription. Affymetrix GeneChip Rat Genome 230 2.0 array was used in this study and the raw image was acquired by scanning the arrays using GeneChip scanner. A series of quality control parameters associated with assay and hybridization performance, such as Probe Array Image Inspection, B2 Oligo Performance, Average Background and Noise Values, Spiking Poly-A Controls (lys, phe, thr, dap), Hybridization Controls (bioB, bioC, bioD, cre) and Percent Present Calling, were examined and all arrays met the Affymetrix quality control parameters and was accepted for further data analysis. Data Analysis was performed using Partek’s Genomics Suite software based on the use of analysis of variance (ANOVA) to obtain differential gene expression data. All data were normalized using the Robust Multi-array Analysis expression statistical analysis (RMA). A minimum of greater than 2 and less than -2 fold change and p < 0.05 thresholds for both up-regulation and down-regulation were selected as the criteria during analyses for the comparison. Classification of the genes was done using DAVID Gene Function Classification Tool (NIAID/NIH)^31^,^32^.

### Animal injection and brain slice staining

Animal experiments were carried out in accordance with the protocol approved by the Institutional Animal Care and Use Committee of Houston Methodist Research Institute. For developing brain study groups of three pregnant Sprague Dawley rats were injected intraperitoneally (i.p.) with SL327 compound (Tocris Bioscience, Bristol, UK) or DMSO control daily for 3 days from embryonic day 18 to 20. SL327 was dissolved in DMSO:PBS = 1:1 and 150 μl volume was injected to reach a dosage of 40 mg/kg. For control equal volume of DMSO:PBS was injected. In the last 2 days the animals were also injected with 100 mg/kg BrdU (Sigma-Aldrich, St Louis, MO, USA) daily. The pups were sacrificed on the day of birth (P0) and the brains were collected for analysis. The dissected brains were postfixed in PFA overnight and transferred to 30% sucrose for two days at 4 °C. Brains were then embedded in OCT compound (Sakura Finetek, Torrance, CA, USA) and frozen on dry ice. Coronal sections were made starting from the olfactory bulb side and the position where the lateral ventricle first appeared was used as the reference. From the reference position continuous sections were made at a thickness of 40 μm with a cryostat (Leica, Chicago, IL, USA). The fourth, sixth and eighth sections were used for immunostaining. Cell counting was done manually in a region that was chosen at the same position based on the nuclei staining. The region size was 300 × 225 μm for BrdU counting, 600 × 450 μm for Pax6 and Sox2 counting, 480 × 360 μm for ASCL1 and DCX counting, 840 × 630 μm for GFAP counting. For staining frozen sections were blocked in 5% BSA in 0.4% Triton X-100 and incubated with primary antibodies overnight at 4 °C. For BrdU staining sections were also boiled in 2N HCl for 30 minutes then cooled down before incubate with the primary antibody. Sections were washed three times with 1x PBS in 0.4% Triton X-100 and incubated with Alexa Fluor–conjugated secondary antibodies (Life Technologies, Grand Island, NY, USA) for 1 h. Sections were washed three times with 1× PBS in 0.4% Triton X-100 and mounted under coverslips using Vectashield with DAPI (Vector Laboratories, Burlingame, CA, USA). Images were taken using either an IX81 inverted fluorescence microscope (Olympus, Tokyo, Japan), or a FV1000 laser scanning confocal microscope (Olympus, Tokyo, Japan). The antibodies stained include anti-Pax6 (Millipore, Billerica, MA, USA), anti-Tbr2 (Abcam, Cambridge, MA, USA), anti ASCL1 (MASH1) (Fisher Scientific, Pittsburgh, PA, USA), anti-DCX, anti-GFAP (Cell Signaling, Danvers, MA, USA), and anti-BrdU (Sigma-Aldrich, St Louis, MO, USA).

### Western blot analysis

Cells were lysed in RIPA buffer (Fisher Scientific, Pittsburgh, PA, USA) in the presence of Xpert protease inhibitor cocktail and Xpert phosphatase inhibitor cocktail (GenDEPOT, Barker, TX, USA). Proteins were separated by 4–15% Mini-PROTEAN TGX precast gel (Bio-rad, Hercules, CA, USA) and transferred to a nitrocellulose membrane. The antibodies tested included the anti-Erk1/2 antibody; anti-phospho-Erk1/2 (Y202/Y204) antibody; anti-Akt antibody; anti-phospho-Akt (S473) antibody; anti-phospho-Akt (T308) antibody; anti-β-catenin antibody; anti-phospho-β-catenin (S33/S37/T41) antibody; anti-FoxO1 antibody; anti-FoxO3a antibody; anti-phospho-FoxO1 (T24) antibody; anti-phospho-FoxO3a (T32) antibody; anti-β-actin antibody. All the antibodies were purchased from Cell Signaling (Cell Signaling, Danvers, MA, USA).

### Statistics

Statistical analyses were carried out using a two-tailed Student’s t-test. Data presented in the graphs are the mean values with the error bars representing the standard deviation (s.d.).

## Additional Information

**How to cite this article**: Rhim, J. h. *et al*. Cell type-dependent Erk-Akt pathway crosstalk regulates the proliferation of fetal neural progenitor cells. *Sci. Rep.* **6**, 26547; doi: 10.1038/srep26547 (2016).

## Supplementary Material

Supplementary Information

Supplementary Information

Supplementary Information

Supplementary Information
