# Supplementary material for: Cell type-dependent Erk-Akt pathway crosstalk regulates the proliferation of fetal neural progenitor cells
Source: Sci Rep. 2016 May 23;6:26547. doi: 10.1038/srep26547 (PMC4876380; doi:10.1038/srep26547)
Supplement: Supplementary Information [file srep26547-s1.pdf]

Cell type-dependent Erk-Akt pathway crosstalk regulates the proliferation of fetal neural progenitor cells

Ji heon Rhim[1,†], Xiangjian Luo[1,†], Dongbing Gao[1], Xiaoyun Xu[1], Tieling Zhou[1], Fuhai Li[1,3], Ping Wang[2,3], Stephen T. C. Wong[1,3], Xiaofeng Xia[1,3,*]

[1]Chao Center for BRAIN, Department of Systems Medicine and Bioengineering, [2]Department of Pathology and Genomic Medicine, Houston Methodist Research Institute, Houston, TX 77030

[3]Weill Cornell Medical College, Cornell University, New York, NY 10065

[†]These authors contributed equally to this work

[*]Correspondence and requests for materials should be addressed to X.X. (xxia@houstonmethodist.org)

FIGURES

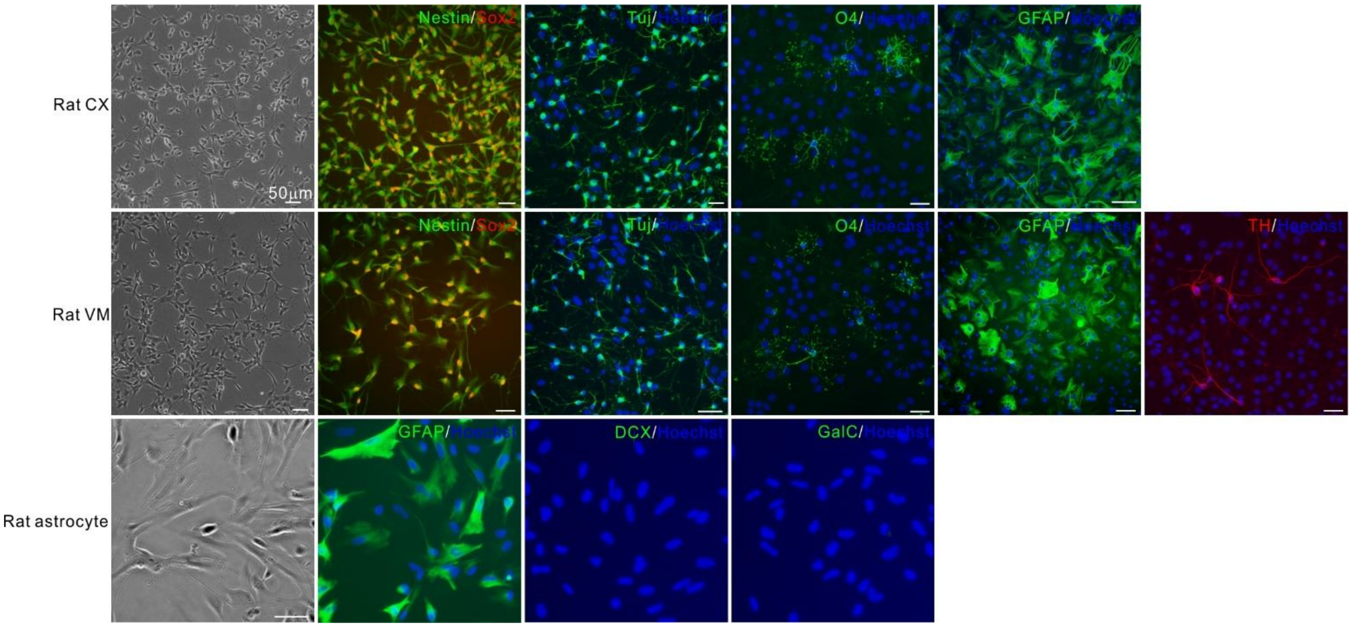

Supplementary Figure 1. The primary fetal NP cells and astrocytes used in the screen.

NP cells were primarily cultured from the E18 embryonic rat cortex (rat CX), and E14 embryonic rat ventral mesencephalon (rat VM). Both cell types exhibit typical neural cell morphology (left bright field images) and homogeneously express NP cell markers Nestin and Sox2. The tripotency of the cells were confirmed by their abilities to differentiation into neurons (Tuj+), oligodendrocytes (O4+) and astrocytes (GFAP+). Cells derived from the ventral mesencephalon region can also be differentiated into dopaminergic neurons (TH+). The control astrocytes were cultured from the E19 rat cortex, the majority of the cells (>80%) are GFAP+, DCX+ neurons and GalC+ oligodendrocytes were not detected.

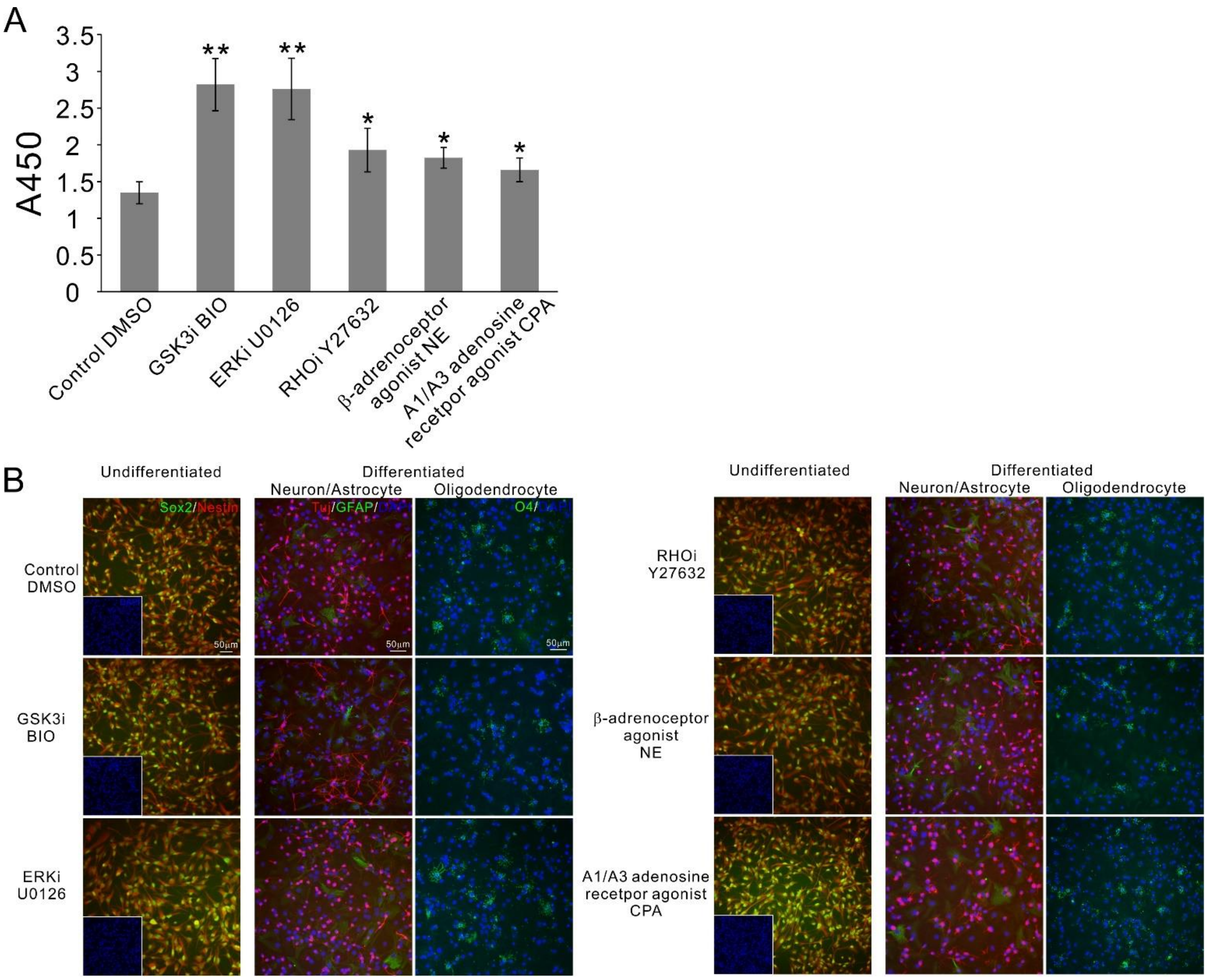

Supplementary Figure 2. Additional characterization of the compound treated fetal NP cells.

(A) Confirmation of the high proliferative capability of the compound treated cells by BrdU cell proliferation assay. Rat CX cells were treated with indicated compound for 3 days and chased with BrdU for 4 hours before the assay. (B) Confirmation of the differentiation capability of the compound treated cell. Rat CX cells were treated with the indicated compound for 3 days. Then the cells were either fixed to test the NP cell identify by staining with the Sox2 and nestin antibodies, or differentiated to test the generation of neuron, astrocytes or oligodendrocytes. For the neuron/astrocyte test, cells were differentiated for 2 weeks and stained with the Tuj and GFAP antibodies, for oligodendrocytes, cells were differentiated for 4 weeks and stained with the O4 antibody.

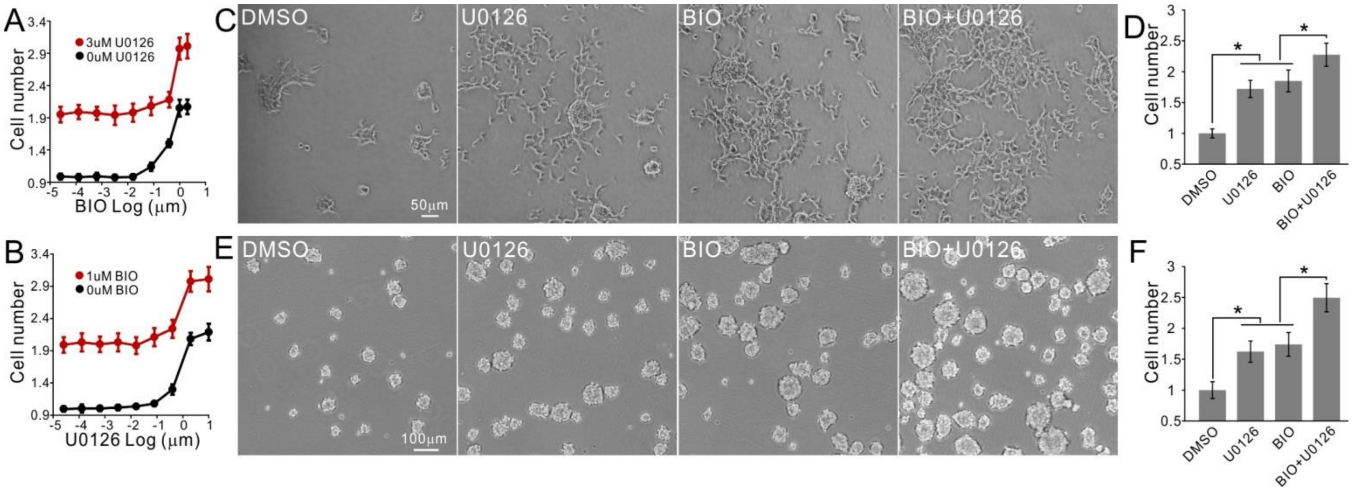

Supplementary Figure 3. ERKi and GSK3i combinatorial effect on fetal NP cell proliferation.

(A) Dose response curves of GSK3i BIO, in the presence or absence of 3 μM ERKi U0126. Cells were cultured as monolayer on laminin coated surface. Mean±s.e.m., n=3. (B) Dose response curves of ERKi U0126, in the presence or absence of 1 μM GSK3i BIO. Cells were cultured as monolayer on laminin coated surface. Mean±s.e.m., n=3. (C) Combinatorial effect of ERKi and GSK3i on the proliferation of fetal NP cells cultured in semi-attached condition on gelatin coated surface. (D) Quantification of the number of semi-attached cells after treatment. Mean±s.e.m., n=4. *, $P<0.05$. (E) Combinatorial effect of ERKi and GSK3i on the proliferation of fetal NP cells cultured as classical neurospheres. (F) Quantification of the number of neurosphere cells after treatment. Mean±s.e.m., n=3. *, $P<0.05$.

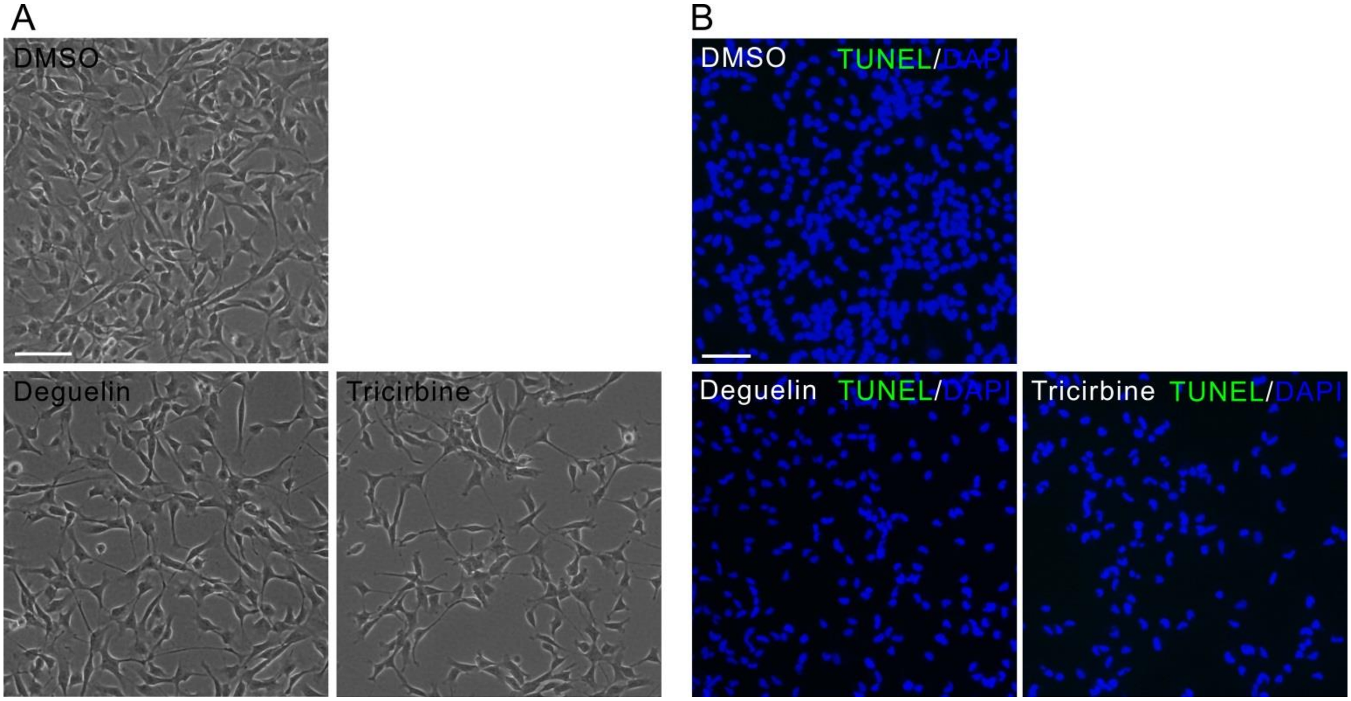

Supplementary Figure 4. Akt inhibitor treated fetal NP cells.

(A) The morphology of Rat CX cells treated with 20 nM Deguelin or 500 nM Tricirbine were the same as the control cells, although the cell numbers were reduced. (B) No apoptosis was detected in the Rat CX cells treated with 20 nM Deguelin or 500 nM Tricirbine.

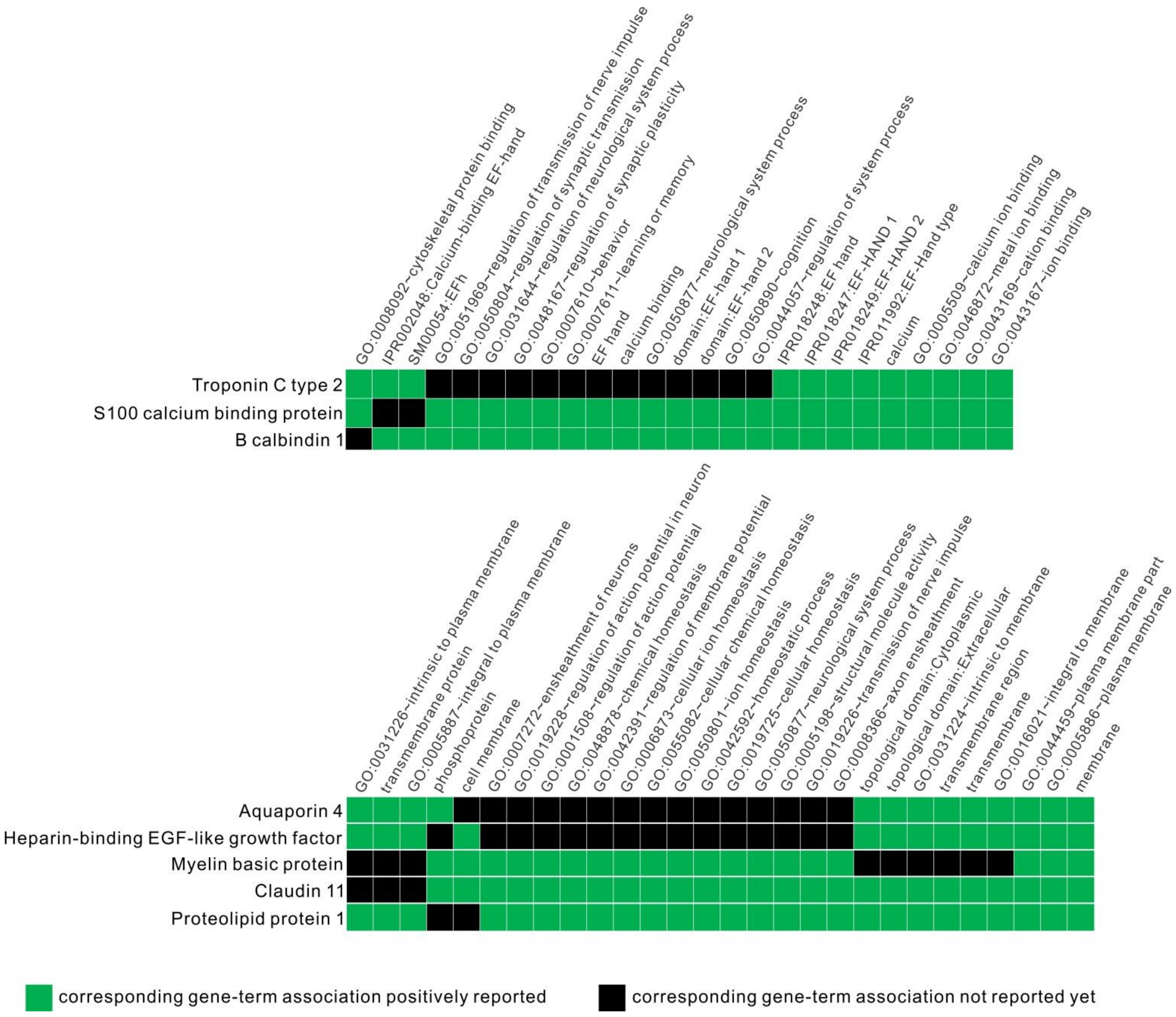

Supplementary Figure 5. Functional classification of the genes changed by more than 2-fold and p<0.05. Two clusters were identified when the classification stringency was set at the lowest. None of the gene function annotations was associated with the function of NP cells. When medium classification stringency was used no cluster could be detected.

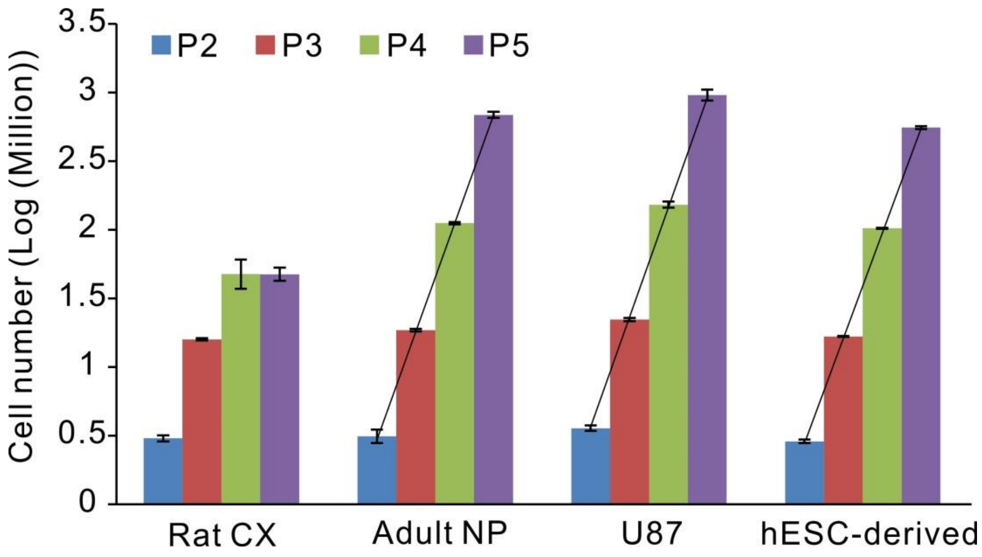

Supplementary Figure 6. Comparison of the cell proliferative capacity . For all the cell types the culture was started by seeding 500,000 cells in a T25 flask, and cells were passaged every 3 days. Total cell numbers were counted in every passage. While the proliferation of Rat CX cells gradually decreased, all the other cell types showed constant cell expanding rate as indicated by the straight line fit ($R>0.9$ for all 3 cell types).

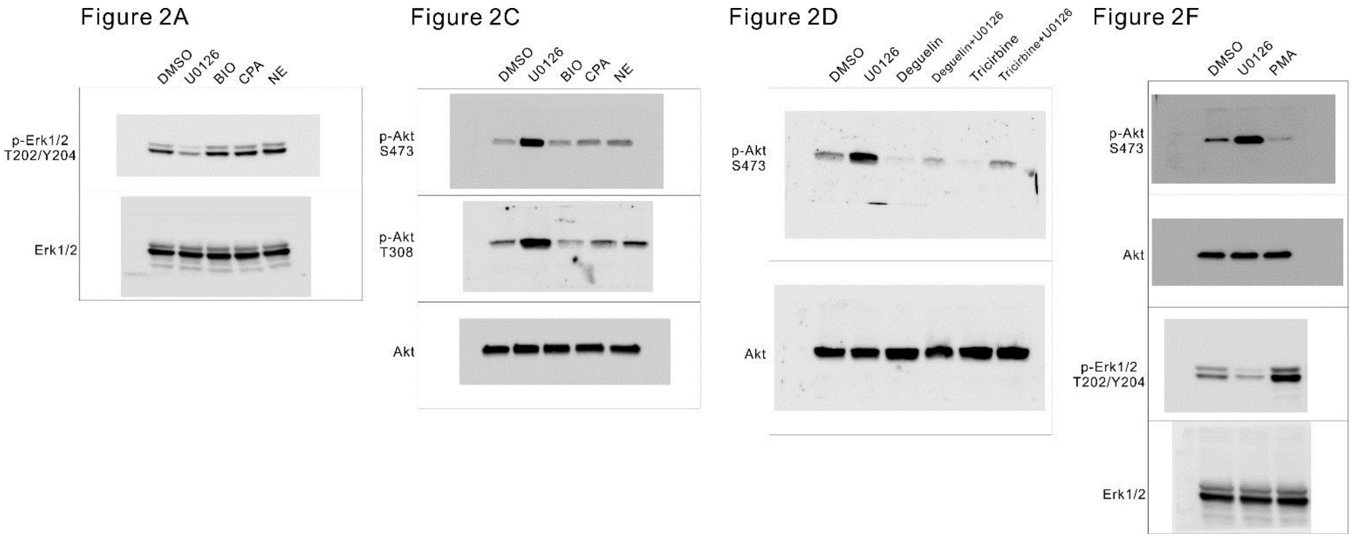

Full length blots for Figure 2

Figure 3A

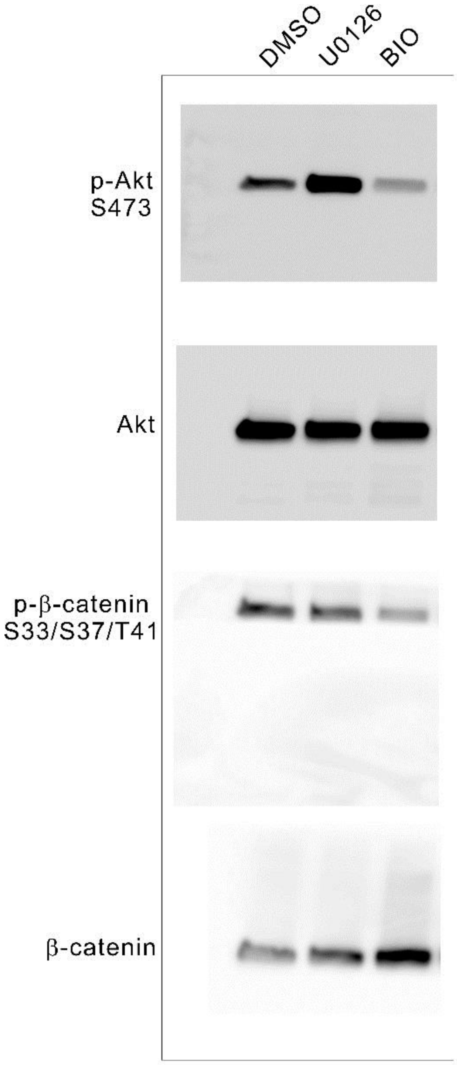

Figure 3B

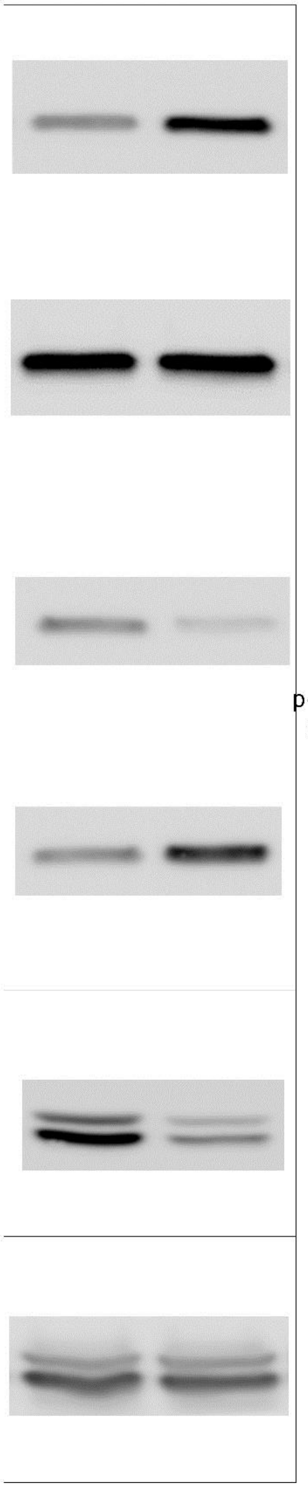

Figure 3C

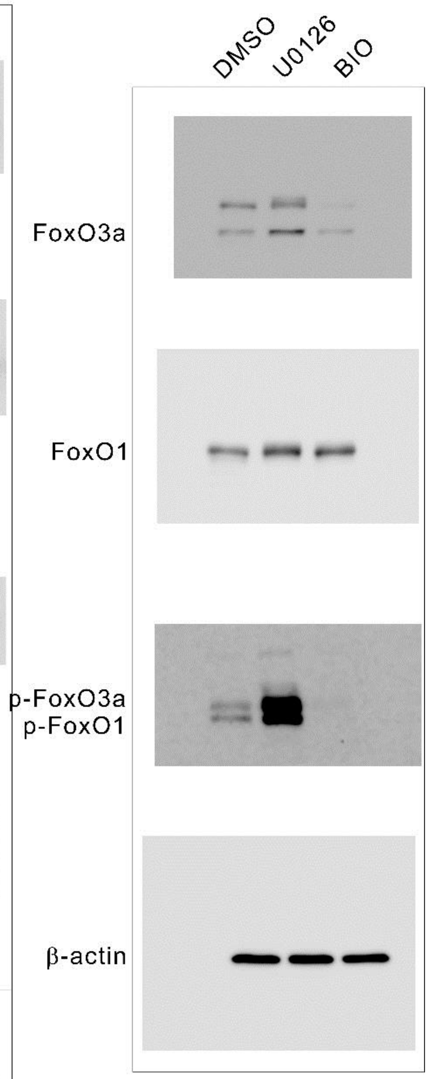

Full length blots for Figure 3

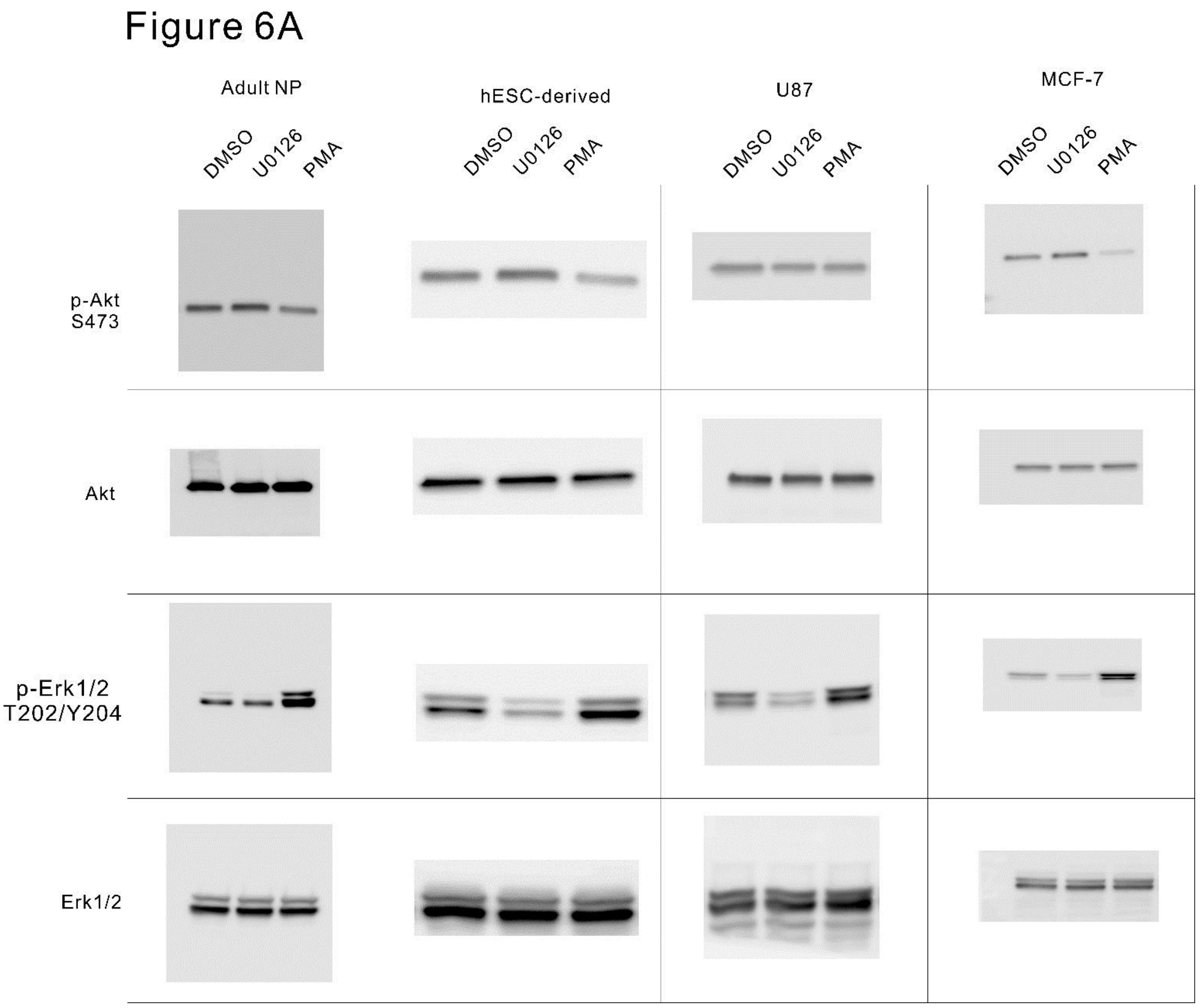

Full length blots for Figure 6

Supplementary Table 1. Primary screening results

(Please see the supplementary Excel file)

Supplementary Table 2. List of the 5 categories of compounds tested in the fetal NP cell proliferation assay

**1. GSK3i**	
1	Kenpaullone
2	SB 216763
3	SB 415286
4	Indirubin-3'-oxime
5	NSC 693868
6	TCS 2002
7	BIO
8	Indirubin
**2. ERKi**	
1	PD 198306
2	PD 98059
3	U0126
4	SL 327
5	FR 180204
**3. RHOi**	
1	Fasudil hydrochloride
2	HA 1100 hydrochloride
3	H-89·2HCl
4	Y-27632·2HCl
**4. Adrenoceptor**	
**Epinephrines**	
1	(-)-alpha-Methylnorepinephrine
2	(-)-Epinephrine bitartrate
3	(ñ)-Epinephrine hydrochloride
4	(ñ)-Norepinephrine (+)bitartrate
5	6-Fluoronorepinephrine hydrochloride
6	EPINEPHRINE BITARTRATE
7	ETHYLNOREPINEPHRINE HYDROCHLORIDE
8	L(-)-Norepinephrine bitartrate
9	NOREPINEPHRINE
10	Racepinephrine HCl
**β Adrenoceptor agonists**	
11	(-)-Isoproterenol hydrochloride
12	(ñ)-CGP-12177A hydrochloride
13	Albuterol hemisulfate
14	Amiodarone hydrochloride
15	BAMBUTEROL HYDROCHLORIDE
16	BRL 37344 sodium

17	CLENBUTEROL HYDROCHLORIDE
18	Dobutamine hydrochloride
19	Dopamine hydrochloride
20	FENOTEROL HYDROBROMIDE
21	Formoterol
22	Formoterol fumarate
23	Isotharine mesylate
24	Isoxsuprine hydrochloride
25	Levalbuterol hydrochloride
26	Metaproterenol hemisulfate
27	Nylidrin hydrochloride
28	PRONETALOL HYDROCHLORIDE
29	R(-)-Denopamine
30	RACTOPAMINE HYDROCHLORIDE
31	Ritodrine hydrochloride
32	Salbutamol
33	Salmeterol
34	SOTALOL HYDROCHLORIDE
35	TERBUTALINE HEMISULFATE
36	Tolazoline hydrochloride
37	Tulobuterol
38	Tulobuterol hydrochloride
39	Xamoterol hemifumarate
**5. Adenosine receptor**	
**A1 Adenosine receptor agonists**	
1	N6-Phenyladenosine
2	N6-Cyclohexyladenosine
3	(S)-ENBA
4	2-Chloro-N6-cyclopentyladenosine
5	GR 79236
6	2'-MeCCPA
7	SDZ WAG 994
8	(ñ)-5'-Chloro-5'-deoxy-ENBA
9	GR 79236X
10	N6-Cyclopentyladenosine
11	R(-)-N6-(2-Phenylisopropyl)adenosine
12	N6-2-Phenylethyladenosine
13	2-Chloroadenosine
14	NECA
**A3 Adenosine receptor agonists**	
15	N6-2-(4-Aminophenyl)ethyladenosine
16	AB-MECA
17	Chloro-IB-MECA
18	IB-MECA

19	2-Cl-IB-MECA
20	HEMADO
21	PARAXANTHINE

Supplementary Table 3. Gene expression microarray analysis of the U0126 maintained fetal rat NP cells. Listed are 39 genes with >2 fold-change and p<0.05 compared to the DMSO treated control cells. Among them only 2 genes were detected with >5 fold-change and p<0.05.

Column #	Column ID	Gene Symbol	Setup p-value U0126 VS DMSO	Fold-Change U0126 VS DMSO
18987	1386911_at	Atp1a2	0.00150736	2.45383
1502	1368945_at	Bmp2	0.00150736	-2.41756
1540	1368983_at	Hbegf	0.00228979	-2.58584
4752	1372195_at	Tnnc2	0.00404317	2.0035
17579	1385036_at	Sncaip	0.00228979	2.15817
601	1368044_at	Scg2	0.0114234	-2.18562
4747	1372190_at	Aqp4	0.00194798	5.1752
9720	1377163_at	Inhbb	0.00228979	2.67659
14651	1382096_at	Cmtm5	0.0063135	2.1029
19345	1387269_s_at	Plaur	0.00697212	-2.08465
30164	1398270_at	Bmp2	0.0063135	-2.26887
5958	1373401_at	Tnc	0.00673994	-2.57399
9126	1376569_at	Klf2	0.00228979	7.01989
2428	1369871_at	Areg	0.00649444	-2.71592
812	1368255_at	Ntm	0.00380677	3.32253
18979	1386903_at	S100b	0.009744	2.16091
18511	1386041_a_at	Klf2	0.00380677	2.1152
16024	1383469_at	Aldh1a3	0.00659896	-3.2607
916	1368359_a_at	Vgf	0.031652	-2.3789
23108	1391032_at	Sez6	0.0139575	2.08186
637	1368080_at	LOC683573, Rgcc	0.00977453	2.7299
6841	1374284_at	Rassf4	0.00745996	2.53916
23669	1391593_at	Rassf4	0.00745996	2.26684
2356	1369799_at	Abat	0.00831502	2.01407
18824	1386637_at	Fgl2	0.0214911	2.22846
4719	1372162_at	Acss1	0.039431	2.01388
9268	1376711_at	Cldn11	0.0195411	2.27277
2758	1370201_at	Calb1	0.0485793	-2.27002
127	1367570_at	Tagln	0.0286546	2.0586
328	1367771_at	Tsc22d3	0.0111509	2.06463
1367	1368810_a_at	Mbp	0.0412499	5.41606
16071	1383516_at	Fgl2	0.0450496	2.59077
134	1367577_at	Hspb1	0.0234176	2.82885
27688	1395794_at	Tpm1	0.0343841	2.32783
25983	1393911_at	Sh3bp4	0.0103316	2.03081
8707	1376150_at	S1pr3	0.0149166	2.19557

24970	1392894_at	Fgl2	0.0377373	2.52617
19188	1387112_at	Plp1	0.0462915	4.15581
5256	1372699_at	Zfp775	0.0187706	2.02251

Supplementary Table 4. Affymetrix GeneChip Rat Genome 230 2.0 array raw data.

(Please see the supplementary Excel file)

Supplementary Movie 1. Time-lapse comparison of control and ERKi treated Rat CX cells. The mitosis events were circled. Images were taken every 15 minutes for about 2.5 days.

(Please see the supplementary video file)
